# Gender and the professional career of primary care physicians in Andalusia (Spain)

**DOI:** 10.1186/1472-6963-11-51

**Published:** 2011-02-28

**Authors:** Ana Delgado, Lorena Saletti-Cuesta, Luis Andrés López-Fernández, Juan de Dios Luna, Inmaculada Mateo-Rodriguez

**Affiliations:** 1 Andalusian School of Public Health, Cuesta del Observatorio 4, 18080 Granada, Spain; 2 Department of Biostatistics. Faculty of Medicine. University of Granada. Avda. del Hospicio, s/n 18071 Granada, Spain

## Abstract

**Background:**

Although the proportion of women in medicine is growing, female physicians continue to be disadvantaged in professional activities. The purpose of the study was to determine and compare the professional activities of female and male primary care physicians in Andalusia and to assess the effect of the health center on the performance of these activities.

**Methods:**

Descriptive, cross-sectional, and multicenter study. Setting: Spain. Participants: Population: urban health centers and their physicians. Sample: 88 health centers and 500 physicians. Independent variable: gender. Measurements: Control variables: age, postgraduate family medicine specialty (FMS), patient quota, patients/day, hours/day housework from Monday to Friday, idem weekend, people at home with special care, and family situation. Dependent variables: 24 professional activities in management, teaching, research, and the scientific community. Self-administered questionnaire. Descriptive, bivariate, and multilevel logistic regression analyses.

**Results:**

Response: 73.6%. Female physicians: 50.8%. Age: female physicians, 49.1 ± 4.3 yrs; male physicians, 51.3 ± 4.9 yrs (p < 0.001). Female physicians with FMS: 44.2%, male physicians with FMS: 33.3% (p < 0.001). Female physicians dedicated more hours to housework and more frequently lived alone *versus *male physicians. There were no differences in healthcare variables. Thirteen of the studied activities were less frequently performed by female physicians, indicating their lesser visibility in the production and diffusion of scientific knowledge. Performance of the majority of professional activities was independent of the health center in which the physician worked.

**Conclusions:**

There are gender inequities in the development of professional activities in urban health centers in Andalusia, even after controlling for family responsibilities, work load, and the effect of the health center, which was important in only a few of the activities under study.

## Background

The number of women in medicine is progressively increasing in numerous countries [1], but they remain unequally distributed among medical specialties [1-3]. Female physicians predominate in general specialties (e.g., pediatrics, psychiatry and family medicine) that are characterized by a much lower salary and prestige [2], a more easily planned work schedule, and a greater interaction with patients in comparison to specialties in which males prevail (e.g., surgery) [1].

Women are underrepresented in healthcare management. In our regional health system, 68% of the health personnel were female in 2007 but only occupied 34% of management posts were held by women (37% in the primary care [PC] sector) [4].

In 2006, 33.8% of male physicians in Catalonia held a management post compared with 18.7% of the females (in the PC sector: 16.9% of males vs. 6.2% of females) [5].

Female physicians are also poorly represented on the governing bodies of scientific societies [6-8] and any presence is frequently in a secondary position [7]. They also appear to face barriers in the leadership of research projects [9] and the publication of scientific papers [7,10-12]. Hence, despite the increased number of female physicians, they do not appear able to develop career-enhancing activities under conditions of equality with their male counterparts [9,13].

The objectives of this study were to determine and compare the professional activities of female and male PC physicians in Andalusian cities and to evaluate the effect of the health center on the performance of these activities. This study is part of a wider project designed to determine the healthcare activities, objective professional achievements and achievement perceptions of male and female physicians in Andalusia.

## Methods

### Descriptive, cross-sectional, and multicenter study

The study population comprised female and male PC physicians working in health centers in the provincial capitals of Andalusia. Inclusion criteria were: same patient list for ≥1 year and utilization of the PC computerized clinical records system. The study sample was obtained by stages, randomly selecting 88 health centers and then randomly selected a number of physicians from each center according to its size (4 physicians each from 8 centers, 5 physicians each from 12 centers and 6 physicians each from 68 centers). A sample of 250 female and 250 male physicians were enrolled in the study (alpha = 5%; 90% power to detect 15% difference between female and male physicians).

The independent variable was physician gender. Personal and professional control variables were: age; postgraduate family medicine specialty (FMS); work load in November 2007, measured as age-adjusted patient list size and mean number of patients/day attended at the surgery; family load, measured as hours/day devoted to housework from Monday to Friday, hours/day devoted to housework on Saturdays and Sundays, and presence or not at home of individuals needing special care (under 15-year-olds, over 65-year-olds and/or disabled individuals); and family situation, categorized either as living alone with/without children or as living in any other domestic situation.

Dependent variables were 24 professional activities, including those that are essential for physicians to advance in their profession in our setting [14]. Four time points/periods were considered for these data a) *date of questionnaire completion*, for data on position as health center manager, accreditation as tutor of residents, or any university teaching position, possession of PhD; b) *previous year*, for data on recycling courses and training activities undergone, health center clinical sessions given, participation as teacher in training activities and membership of scientific societies; c) *previous 5 years*, for data on positions as principal investigator and collaborating investigator, authorship of original articles and other types of publication in scientific journals, authorship of books or book chapters, authorship or co-authorship of scientific papers presented at congresses; participation as speaker at congresses, membership of congress scientific or organizing committees; and d) *entire professional life*, for data on membership of governing bodies of scientific societies and medical associations and participation in scientific society and national and regional health authority working groups.

Data were gathered from three sources in different time periods, as follows. *Between December 2007 and May 2008*, self-administered questionnaires on personal and professional data were administered, after first telephoning health center managers to obtain their cooperation in delivering the questionnaires and in reminding the physicians to complete and mail them; completed questionnaire were collected by hand when necessary. *Between February and October 2008*, data were gathered from the regional health authority by telephone on: health center managers and size of the patient list of physicians enrolled in the study and their n° patients/day (including data for non-respondents). *In October 2008*, data were obtained from the corresponding family medicine teaching centers on: health centers accredited for resident training, physicians accredited as tutors of residents, and physicians with residents assigned in 2007 (including non-respondents).

### Statistical Analysis

Data were expressed as means ± standard deviation (SD), and bivariate analyses were performed using the chi-square and Student's t tests. Multilevel logistic regression analysis were then performed, considering the GP as first level and the health center as second level and calculating odds ratios (ORs) for the relationship between gender and professional variable adjusted for the control variables. The aim of the multilevel analysis was to evaluate the effect of the health center on dependent variables by calculating the intraclass correlation coefficient (ICC), with a higher ICC signifying a greater effect of the center on the activity. P ≤ 0.05 was considered significant (two-tailed tests). We also report relationships at the limit of significance (p < 0.10). STATA 10.1 was used for the data analyses.

## Results

Responses were received from 368 PC physicians (73.6%): 182 (71.7%) of the females and 186 (75.6%) of the males. The mean age of the respondents was 49.1 ± 4.3 yrs for the females versus 51.3 ± 4.9 yrs for the males (p < 0.005). After exclusion of the non-respondents, the statistical power of the study was reduced to 60.3%.

Comparison of the four personal variables obtained in all enrolled physicians showed no differences between respondents and non-respondents except that health center managers were more likely than non-managers to be respondents (Table 1).

**Table 1:** Questionnaire response rate according to four sample characteristics

Variables	Sample (N = 500)	Respondents	Non-respondents	P
**Physician gender**				
Female	254	182 (71.7%)	72(28.3%)	0.32
Male	246	186 (75.6%)	60 (24.4%)	

**Tutor of FMS residents**				
No	372	267 (71.8%)	105 (28.2%)	0.11
Yes	128	101 (78.9%)	27 (21.1%)	

**Heath Center Manager**				
No	462	331 (71.6%)	131 (28.4%)	0.001
Yes	38	37 (97.4%)	1 (2.6%)	

**Health Center accredited for FMS training**				
No	264	199 (75.4%)	65 (24.6%)	0.34
Yes	236	169 (71.6%)	67 (28.4%)	

FMS: Family Medicine Specialty

As shown in Table 2, the female physicians were younger, more frequently possessed the family medicine specialty, devoted more hours to housework, and were more likely to live alone in comparison to the males. There were no gender differences in work load variables.

**Table 2:** Comparison by gender of control variables

Quantitative Variables	Female physicians Mean (SD)	Male physicians Mean (SD)	P
Age	49.1 (4.3)	51.3 (4.9)	<0.001
Hours housework Monday to Friday	2.9 (3.1)	1.1 (1.0)	<0.001
Hours housework weekends	4.1 (2.9)	1.9 (1.6)	<0.001
Patient quota	2055 (224.8)	2041 (304.1)	0.65
Patients on demand/day	36.5 (8.6)	36.4 (9.3)	0.86

**Qualitative Variables**	**Female Physicians N (%)**	**Male Physicians N (%)**	**P**

Family Medicine Specialty	80 (44.2%)	62 (33.3%)	0.02
Lives alone			
with children	11 (6.1%)	5 (2.7%)	0.02
without children	18 (9.9%)	4 (2.2%)	
Individuals in household needing special care	77 (42.3%)	79 (42.5%)	0.97

Table 3 depicts the crude and adjusted ORs for the relationships between gender and professional activities. The adjusted OR was significant for health center manager, recycling courses, clinical sessions led at the health center, collaborating investigator, author of original article, author of book/chapter, co-author of congress paper, member of governing body of scientific society, member of governing body of medical association, and member of public health service working group (p < 0.05), while it was at the limit of significance for participation as teacher, congress participant, and congress speaker (p < 0.10). 10 of the 24 professional activities studied and at the limit of significance (0.05 < p < 0.10) in a further 3: all of these 13 activities were performed less frequently by female physicians after adjusting for family responsibilities (3 variables), family situation, and work load (2 variables), among others.

**Table 3:** Female (182) and male (186) physicians' participation in professional activities.

			Unadjusted Model	Adjusted Model	
				
Professional Activities	Female physicians (Reference category) N (%)	Male GPs (Risk category) N (%)	OR (95% CI)	OR (95% CI)	ICC
Health center manager	9 (4.9)	28 (15.1)	3.41 * (1.56; 7.44)	4.69 * (1.83;12.03)	<0.001

Accredited FMS tutor	51 (28.0)	50 (26.9)	1.24 (0.58; 2.62)	1.87 (0.67;5.17)	0.77

Assigned FMS resident	40 (22)	42 (22.6)	1.33 (0.66; 2.69)	1.72 (0.72;4.13)	0.67

University position	6 (3.3)	10(5.4)	1.77 (0.59;5.30)	2.64 (0.63;11.09)	0.31

PhD	30(16.5)	40(21.5)	1.39 (0.82; 2.35)	1.33 (0.71; 2.51)	0.01

Recycling courses	70 (38.5)	96 (51.6)	1.82* (1.15; 2.87)	2.17* (1.25; 3.76)	0.16

Attendance at training courses	147 (80.8)	155(83.3)	1.19 (0.69;2.02)	1.42 (0.74;2.72)	<0.001

Clinical sessions led at Health Center	114 (63.3)	122(67.8)	1.27 (0.78; 2.07)	2.58* (1.40; 4.76)	0.22

Participation as teacher	68 (37.4)	80 (43.0)	1.30 (0.84; 2.02)	1.68** (0.99; 2.83)	0.08

Member of Scientific Society	126(69.6)	132(71.4)	1.09 (0.69;1.74)	1.55 (0.86; 2.79)	0.05

Principal investigator	16 (8.8)	22(11.8)	1.35 (0.66; 2.76)	1.06 (0.44; 2.52)	0.19

Collaborating investigator	67(36.8)	98(52.7)	1.97* (1.28; 3.05)	2.50* (1.49; 4.19)	0.04

Author of original article	16(8.8)	39(21)	2.82* (1.49; 5.35)	2.99* (1.43; 6.23)	0.70

Author of other type of article	18 (9.9)	24 (12.9)	1.37 (0.68;2.77)	1.67 (0.74; 3.77)	0.27

Author of book or chapter	20 (11)	37(19.9)	2.31* (1.20;4.42)	3.57* (1.61;7.90)	0.20

Principal author of congress paper	24(13.2)	27(14.5)	1.10 (0.57; 2.14)	1.62 (0.72;3.64)	0.33

Co-author of congress paper	40(22)	65(35)	2.11* (1.24;3.59)	2.61* (1.37; 4.97)	0.27

Congress participant	119(67.2)	133(74.3)	1.4 (0.89; 2.24)	1.6** (0.94; 2.79)	0.01

Congress speaker	21(11.5)	40 (21.5)	2.16* (1.19;3.93)	1.97** (0.99; 3.89)	0.08

Member of congress scientific or organizing committee	16 (8.8)	21(11.3)	1.35 (0.67; 2.72)	1.29 (0.58; 2.89)	0.08

Member of governing body of scientific society	7 (3.9)	18(9.8)	2.83* (1.12; 7.18)	3.05* (1.06; 8.74)	0.13

Member of governing body of medical association	2 (1.1)	9 (4.8)	5.11 (0.99;26.27)	15.61* (1.19;205.05)	0.19

Member of scientific society working group	34 (18.7)	54 (29.0)	1.79* (1.09; 2.94)	1.58 (0.88; 2.82)	0.01

Member of Public Health Service working group	82 (45.1)	102(54.8)	1.54 (0.99; 2.38)	1.85* (1.11; 3.08)	0.09

Crude and adjusted Odds Ratios with 95% Confidence Intervals. Intraclass Correlation Coefficient (ICC) of Health Center.

FMS: Family medicine Specialty

** 0.05 < p < 0.10

* p < 0.05

The ICC was virtually 0% for health center management and course attendance. The ICC was >0.50 for assignment of resident, authorship of original articles, and resident tutorship.

## Discussion

The response rate in this study was very acceptable for a self-administered questionnaire, and respondents only differed from non-respondents in the higher proportion of center managers responding; these represented only 7.6% of the sample and had been personally contacted about the questionnaire.

The female physicians were younger and were more frequently specialized in family medicine, while there were no gender differences in patient list size or mean n° patients/day, as also found in a study of 32 European countries [15]. Although there was no difference in cohabitation with dependent individuals, the females devoted more than double the time to housework and were more than three times more likely to live alone with children in comparison to the males, reproducing the situation in the general population [16,17]. Hence, the higher professional qualifications of female physicians in comparison to the general population do not appear to have improved their conditions of inequality in domestic tasks. Interestingly, females comprised 68% of our regional health service healthcare professionals in 2007, as noted in the Introduction, but made 74% of the requests for permission to care for a relative (excluding birth-related requests) [4].

After adjusting for age, family medicine specialty, care load, family load, and family situation, the participation of female physicians was significantly less frequent in 13 of the 24 activities in comparison to the males. In all activities, except for congress participation as a speaker, the adjusted odds ratio was higher than the crude odds ratio, i.e., the gender inequality in the professional activities was more marked after adjustment for work and family loads.

Only 5% of female physicians were health center managers in comparison to 15% of male physicians; mirroring the situation observed elsewhere in Spain [5,9] and other countries [1,18].

The higher proportion of female physicians with the family medicine specialty does not translate into differences in accreditation as resident tutor, suggesting that women do not take full advantage of their superior training. After postgraduate training, male and female physicians attend the same number of courses but the females are less likely to participate in recycling activities that entail leaving their surgery for weeks at a time. Moreover, female physicians less frequently conduct sessions for their teams or take on a teaching role.

There was no gender difference in participation in scientific societies or medical associations (compulsory), but female physicians are less likely to sit on the governing body of these organizations, as found by other studies [1,6,7]. According to one report [9], although 45% of physicians are female, they only occupy 33% of management posts. In the present study, the participation of female physicians was similar to that of males in working groups of scientific societies but lower in national and regional health authority working groups.

There was no significant gender difference in work as principal investigator, likely due to the small numbers involved, but the female physicians were less frequently collaborative investigators. Likewise, there was no difference in being the first author of scientific papers, but female physicians were less frequently co-authors. It is possible that the females focused more on leading projects and authoring papers than on collaborating with colleagues. An alternative interpretation is that they are less frequently included in research led by others, as claimed by female physicians in a previous study [19]. The male physicians attended more scientific congresses and more frequently presented papers, as observed in the congresses of other scientific societies [8], although was no gender difference in congress committee participation.

The female physicians published fewer original articles and books, but there was no gender difference in other types of publication. A review of four Spanish medical journals found that 71% of the first authors were male [11]. The proportion of female authors in the publication of original articles is increasing and varies according to the specialty, but data published by two reviews, one of six US journals [10] and another of six UK journals [20], suggest that a gender gap persists, more markedly for the last (senior) author. The lesser career progress of female physicians has been attributed to their inferior productivity, but adjusting for publications and work load, they continue to occupy lower professional positions [21,22].

Jiménez-Rodrigo [12] summarizes the complex causes of gender inequality in the publication process, including: the underrepresentation of females on editorial committees; the role of influence networks in citations and peer reviews, more favorable for men; and the selection of "softer" research subjects by women, with different methodological criteria, which are frequently less readily accepted by the scientific community.

There were no significant gender differences in the possession of a PhD or university position, although a small number of the physicians fulfilled this condition. Nationally, only 26% of physicians with university teaching positions were female in the academic year 2007-2008 [23].

According to the ICC findings, the health center had a significant impact on the tutorship and assignment of residents, which took place in 41 health centers (containing 236 of the physicians). The health center also influenced the authorship of original articles (including co-authorship), with the 55 authors grouped in a few centers (0.70 ICC). Nevertheless, a gender difference in this activity remained after controlling for the effects of the health center. However, due to our cross-sectional study design, we cannot know whether this high ICC implies cause or effect, i.e., whether physicians who publish as a group together in certain health centers or whether conditions in some centers favor research activity.

The health center had no influence on differences observed in the remaining activities studied (low ICC values).

It has been proposed that professional gender inequalities will diminish with the growing proportion of females in the medical profession (cohort or generation effect) [24]. However, although all categories of the professional hierarchy are feminizing, there is evidence that women remain disadvantaged in their access to the highest levels [24,25]. Thus, it has been reported the leadership levels now achieved by female physicians are no higher than they were some decades ago, and that this phenomenon cannot be explained by a so-called "period" or "environmental" effect [18].

Riska [26] brings together two mechanisms by which gender inequalities in medical careers are maintained. One relates to the variation in decision-making due to gender differences in socialization, based on sex role theory. The other involves structural barriers, based on the concept of the "glass-ceiling" [27,28], i.e., an invisible barrier to the advancement of women (in general) to the highest posts, formed by external obstacles (organization structure, organizational culture, and gender stereotypes), internal obstacles (gender identity, achievement motivation, and personality aspects), and interactive obstacles.

Studies have identified different categories of obstacle for female physicians, including rigidity of the organization and professional career, sexual discrimination, psychological barriers, career motivation and family responsibilities [29,13,30]. These responsibilities and the difficult balance between family tasks and work were found to be the greatest impediment to career advancement in a North America study [19] and were reported to diminish the interest of female physicians in their career [31]. Aspects of the interaction between family and work roles were reported to be causes of dissatisfaction among female medical faculty in the USA [30]. There is some evidence that male and female expectations of the work-life balance are changing, with both genders desiring its improvement [32]. One US medical school [33] found that career difficulties for female physicians augmented with the larger number of children they had, although this finding was not replicated in a subsequent study in the same setting [25] or in the United Kingdom [34]. It has been claimed that gender inequalities will no longer pose a problem when female physicians do not have to choose between family responsibilities and professional status [35]. The Association of American Medical Colleges has published the most effective measures for improving the success and leadership of female physicians and reducing inequalities [36].

Study limitations include the cross-sectional design, preventing study of the direction of the relationships found, and the restriction of health centers and PC physicians to the urban setting, preventing extrapolation of results to PC physicians as a whole. In addition, the statistical power of the study was reduced to 60% after excluding non-respondents. However, the study has internal validity, since the main objective was to compare professional activities by gender. There may also have been information bias, since all except for six of the analyzed activities were self-reported, although this risk is limited by the use of the same data source (questionnaire) for both genders.

Two major study strengths are the inclusion of some family variables and the multi-level analysis. As a result, we were able to determine the association of GP gender with professional activities after controlling for the effects of the family and health center settings.

Longitudinal studies are required to improve our knowledge of this issue, analyzing the professional performance of each gender, identifying barriers, enhancing the development of female leaders and supporting their needs [1,13,30].

There is also a need for qualitative studies that address the meaning and complexity of this phenomenon for female and male physicians, integrating this information with quantitative data.

## Conclusions

These results strongly indicate that female physicians are disadvantaged in Andalusia. Gender inequalities were found in the development of professional activities of physicians in urban health centers, even after controlling for family responsibilities and the effect of the center, which was important in only a few of the activities studied. This situation of disadvantage is likely to have a negative effect on the professional career of female physicians.

## Competing interests

The authors declare that they have no competing interests.

## Authors' contributions

AD conceived of the study, wrote the manuscript and made substantial contributions to the design of the study.

LSC performed the data collection, contributed to the interpretation of data, and wrote the manuscript.

LALF conceived of the study, contributed to the design and interpretation of data, and commented on the manuscript.

JDL conceived of the study, contributed to the conception and design of the study, and performed the statistical analyses.

IMR conceived of the study, supervised the design and commented on the manuscript.

All authors have read and approved the final manuscript.

## Pre-publication history

The pre-publication history for this paper can be accessed here:

http://www.biomedcentral.com/1472-6963/11/51/prepub
